# Metabolism and Functions of Inositol Pyrophosphates: Insights Gained from the Application of Synthetic Analogues

**DOI:** 10.3390/molecules25194515

**Published:** 2020-10-02

**Authors:** Stephen B. Shears, Huanchen Wang

**Affiliations:** Inositol Signaling Group, Signal Transduction Laboratory, National Institute of Environmental Health Sciences, National Institutes of Health, Research Triangle Park, NC 27709, USA; huanchen.wang@nih.gov

**Keywords:** metabolism, homeostasis, phosphate, cell biology, energy

## Abstract

Inositol pyrophosphates (PP-InsPs) comprise an important group of intracellular, diffusible cellular signals that a wide range of biological processes throughout the yeast, plant, and animal kingdoms. It has been difficult to gain a molecular-level mechanistic understanding of the actions of these molecules, due to their highly phosphorylated nature, their low levels, and their rapid metabolic turnover. More recently, these obstacles to success are being surmounted by the chemical synthesis of a number of insightful PP-InsP analogs. This review will describe these analogs and will indicate the important chemical and biological information gained by using them.

## 1. Introduction

**Figure 1:**
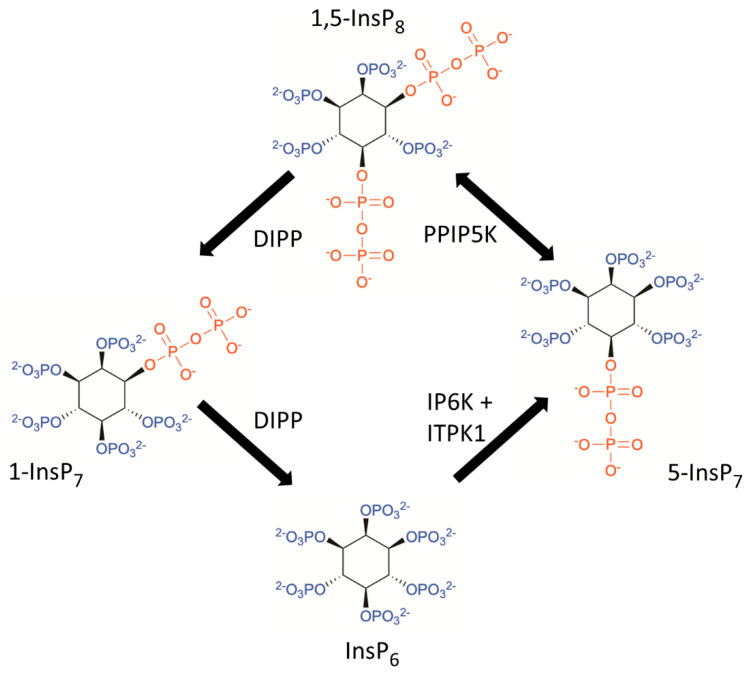
The inositol pyrophosphate (PP-InsP) metabolic pathway. This graphic incorporates a proposal [1,2] that the pathway for 1,5-InsP_8_ turnover principally comprises a cyclical interconversion of InsP_6_, 5-InsP_7_, 1,5-InsP_8_, and 1-InsP_7_. ITPK1 = inositol 3,4,5,6-tetrakisphosphate 1-kinase; IP6K = inositol hexakisphosphate 5-kinase; PPIP5K = diphosphoinositol pentakisphosphate 1-kinase (in addition to hosting a self-contained kinase domain, this class of enzyme also contains a phosphatase domain that possesses 1,5-InsP_8_ 1-phosphatase activity [2,3,4]); DIPP = diphosphoinositol polyphosphate phosphatase.

The inositol pyrophosphates (PP-InsPs), particularly 1-InsP_7_, 5-InsP_7_, and 1,5-InsP_8_ (Appendix A; Figure 1) are highly ‘energetic’ and diffusible signaling molecules that play important roles in many cellular processes throughout the eukaryotic kingdoms, particularly with regards to phosphate and bioenergetic homeostasis, both of which can have profound metabolic and physiological consequences [1,2,3,4,5,6,7,8]. Indeed, it is hard to overstate the significance of dovetailing any signal transduction cascade with metabolic circuitry. All biological programs place bioenergetic demands upon an organism; its ability to adapt to environmental challenges, i.e., its long-term survival, is reliant upon signaling cascades exerting dynamic control over the uptake, storage, and utilization of various metabolic fuels [9]. Our understanding of the many roles that PP-InsPs play in these processes has been especially well-served in recent years by the contributions that organic chemists have made in synthesizing insightful PP-InsP analogs. Here, we review the nature of these tools and the knowledge gained from their use.

## 2. PP-InsP Synthesis and Metabolism

Considerable attention has been given to two classes of kinases that work together to generate PP-InsPs (Figure 1): IP6Ks (see [6]), which until very recently were considered solely responsible for phosphorylating InsP_6_ to 5-InsP_7_, and the PPIP5Ks (see [2]), which interconvert 5-InsP_7_ with 1,5-InsP_8_. However, it has been enigmatic that orthologs of IP6Ks are not expressed in plants. This conundrum was recently resolved with the determination that the ‘missing’ InsP_6_ kinase activity in plants is expressed by ITPK1s [10,11,12], a class of enzyme that had been originally characterized as active Ins(3,4,5,6)P_4_ 1-kinases [13]. Human ITPK1 also expresses this InsP_6_ kinase activity in vitro [10], but it has yet to be demonstrated if it is sufficient in vivo to be considered a genuine, alternate source of 5-InsP_7_ synthesis. Incidentally, an ITPK1 ortholog has not been identified in any yeast or insect genome.

The major pathway for net catabolism of 1,5-InsP_8_ back to InsP_6_ in animal cells (see Figure 1) is proposed to occur by successive 5-β-phosphatase and then 1-β-phosphatase activities of the diphosphoinositol polyphosphate phosphatases (DIPPs), although the 1-InsP_7_ (Figure 1) is only a short-lived intermediate [1,14]. In *Saccharomyces cerevisiae*, this pathway of 1,5-InsP_8_ turnover is reinforced through its hydrolysis by an additional 5-phosphatase activity catalyzed by Siw14 [15,16]. No animal orthologs of Siw14 have been identified to date.

## 3. Metabolically Stable Analogues of 5-InsP_7_

A number of studies have explored the biological activities of the PP-InsPs by pharmacological and/or genetic intervention in the metabolic pathway described by Figure 1 (see [6,8] for recent, authoritative reviews). However, the blockage of IP6K activity by itself cannot distinguish the actions of 5-InsP_7_ from those of InsP_8_, since the synthesis of both is reduced. The inhibition of PPIP5K activity is not a specific strategy either; both InsP_8_ and 1-InsP_7_ synthesis is compromised, and there is a significant elevation in 5-InsP_7_ levels [17,18,19]. Thus, genetic and pharmacological experiments do not necessarily attribute a particular phenotype to the action (or loss of action) of one specific PP-InsP isomer. This is a situation in which metabolically stable PP-InsP analogs can be helpful, particularly with regards to biochemical and cellular assays, in which the natural PP-InsPs have a short half-life due to their inherently fast turnover rate [20,21]. Another application for such research tools is to distinguish between the two molecular mechanisms by which protein function can be modulated by the PP-InsPs: allosteric regulation upon ligand binding, versus covalent regulation by non-enzymic phosphotransfer [1,6,7,8,22]. The latter cannot be mimicked by metabolically stable PP-InsP analogs.

**Figure 2:**
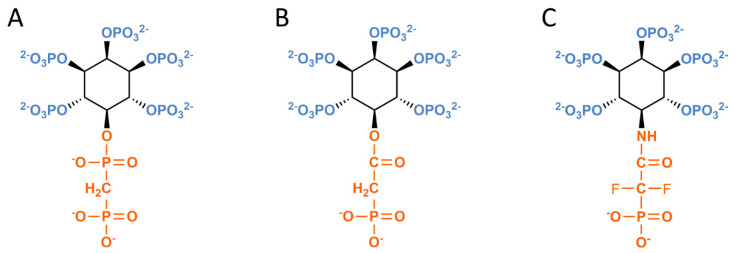
Metabolically stable bioisosteres of 5-InsP_7_. This figure shows synthetic analogs of 5-InsP_7_ in which the phosphoanhydride group is replaced with either (**A**) methylene-bisphosphonate (PCP), (**B**) phosphonoacetate (PA), (**C**) phosphonodifluoroacetamide.

Two teams of researchers have separately pioneered the development of metabolically stable PP-InsP molecules: Barry Potter’s UK group and Dorothea Fiedler’s laboratory, which is now based in Germany. These reagents (Figure 2; Appendix A) were first described in 2012–2013 [23,24]. Fiedler and colleagues synthesized methylene-bisphosphonate (PCP) analogs of 5-InsP_7_ in which a methylene group was substituted for the bridging oxygen of the phosphoanhydride moiety (Figure 2A and [24]). Potter’s group replaced the bisphosphate of 5-InsP_7_ with phosphonoacetate (PA) [23]. Subsequently, the PA- analog of 1,5-InsP_8_ and PCP-analogues of 1-InsP_7_ and 1,5-InsP_8_ were also synthesized [25,26,27].

The authors of the latter studies have referenced earlier work [28] that notes the replacement of an oxygen atom with a methylene group leads to a reduction in the acidity of the phosphonic acid when compared to the phosphoric acid. The pKa values for the second deprotonations are such that the phosphonate will be less strongly ionized at physiological pH compared to the phosphate, potentially leading to a decreased affinity for protein receptors [28]. Despite this caveat, several significant discoveries have emerged from the application of these analogs, and these advances are described in the Sections below.

Potter group’s recently synthesized a new analog in which the phosphonate CH_2_ group was replaced by a more electronegative CF_2_, thereby increasing the acidity of the moiety (Figure 2C) and potentially narrowing the difference in pKa values compared to the natural diphosphate [28]. Since a difluoroacetate ester would be very labile to hydrolysis, a stable amide linkage was used (Figure 2C and [29]).

## 4. Bioisosteres and the Discovery of a PP-InsP Capture Site in the Kinase Domain of PPIP5K

To enforce the tight substrate specificity of the PPIP5Ks for 5-InsP_7_ and, to a minor extent InsP_6_, ligand entry into the catalytic pocket is guarded by substantial architectural and electrostatic constraints [30]. This situation suggests ligand is only admitted when it is presented in the appropriate orientation, i.e., a direct hit. Could that limit the enzyme’s ability to attract ligand that rotates and diffuses randomly through the bulk phase? For an illustrative analogy, consider the frustratingly customary outcome of a classic carnival game: the high failure rate when attempting to throw a table tennis ball into the narrow neck of a distant goldfish bowl.

**Figure 3:**
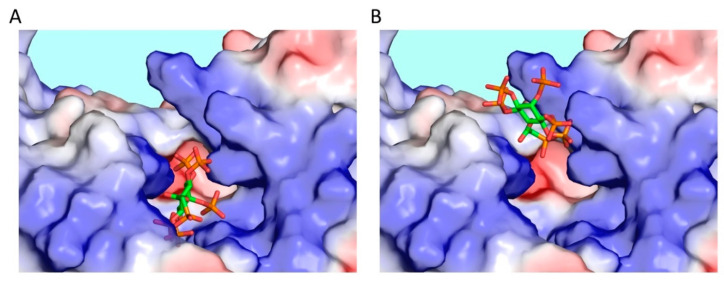
PPIP5K^KD^ has two substrate binding sites. X-ray crystallographic analysis of PPIP5K^KD^/5-PA-InsP_7_ crystal complexes uncovered two mutually exclusive ligand binding sites: (**A**) catalytic pocket; (**B**) substrate capture site. The protein is depicted in surface plot format, in which the intensity of the coloration (blue = positive, red = negative) corresponds to the degree of electrostatic polarization. The ligand is shown in stick form: carbon is green, phosphorous is orange, and oxygen is red.

Our X-ray analysis of 5-PA-InsP_7_ soaked into crystals of the kinase domain of PPIP5K (i.e., PPIP5K^KD^) helped to uncover a near-unique mechanism by which this enzyme activity facilitates substrate access to the active site: two ligand-binding positions were observed [31]. Both ligand-binding sites cannot be occupied by substrate simultaneously, due to steric clashing. Thus, we reasoned that the crystal complexes comprised a mixture of two separate, but mutually exclusive ligand configurations. One of the binding sites for 5-PA-InsP_7_ is near superimposable upon that occupied by 5-InsP_7_ in the catalytic pocket (Figure 3A). A second binding site lies approximately 8 Å away, closer to the surface of the protein (Figure 3B). We propose the latter enhances substrate capture from the bulk phase, prior to its delivery into the catalytic pocket. We have described this phenomenon as a “catch-and-pass” reaction mechanism [31]. In an earlier study, we could not detect 5-InsP_7_ substrate in the capture site [30]. Perhaps a slight difference in the degree of the positive charge of 5-InsP_7_ vis-a-vis its synthetic analog is responsible for the different behavior. We presume that the delivery of natural substrate is more efficient compared to 5-PA-InsP_7_, and hence the natural ligand occupies the capture site too transiently to yield suitable electron density.

The 8 Å journey that 5-InsP_7_ takes when moving between its two binding sites has been modeled by the application of molecular dynamics simulations [32]. This was how we established the role of Glu192 as a molecular ratchet that moves towards the space vacated by the substrate. That is, the Glu192 electrostatically enforces one-way substrate delivery [32]. The concept that this residue is catalytically important has been verified experimentally—a Glu192Gln mutation dramatically reduces kinase activity, presumably by compromising substrate transfer into the active site [31].

The new phosphonodifluoroacetamide analog (Figure 2C) has also been soaked into crystals of PPIP5K^KD^ and found to closely mimic the occupation of the catalytic pocket by 5-InsP_7_ (Figure 2C and [29]); the capture site was not occupied. Interestingly, there is a Mg^2+^ ion that makes contact with 5-InsP_7_ in its crystal complex with PPIP5K^KD^, but the metal is missing from the complex with the phosphonodifluoroacetamide analog. Therefore, it was suggested that this new analog might be particularly useful for studying those PP-InsP/protein interactions that are Mg^2+^-independent [29]. As it happens, there may be many such interactions: it has been noted by others that, to date, Mg^2+^ is missing from all of the published non-catalytic protein/PP-InsP complexes that have been captured by x-ray crystallographic analyses [33].

The analysis of a crystal complex of PPIP5K^KD^/1,5-PA-InsP_8_ showed that this particular analog of the PPIP5K enzymatic product also occupies both ligand binding sites [25]. This observation raises the possibility that the product escapes from the catalytic pocket via the same capture site. In contrast, in corresponding crystal complexes that contain either 1,5-PCP-InsP_8_ or 5-PCP-InsP_7_, these ligands were only observed in the catalytic pocket [25,27], just as is the case with the natural substrates [30,34]. Nevertheless, the PA-version was more informative because it identified the capture site. This comparison underscores the scientific value of probing biological systems with a variety of different analogs.

## 5. The Positional Specificity of DIPPs: Information Gained from PCP-InsPs

The DIPPs show a high rate of hydrolytic activity towards all PP-InsPs [14], but there is a puzzling aspect to the enzymes’ positional specificity. Biochemical assays have indicated that the 5-β-phosphate is preferentially cleaved from 1,5-InsP_7_ [14]. Despite that, these enzymes show a greater preference for the 1-β-phosphate of 1-InsP_7_ compared to the 5-β-phosphate of 5-InsP_7_ [14,35]. Moreover, PCP analogs have been useful in validating the enzyme’s preference for 1-InsP_7_ over 5-InsP_7_. PCP analogs are not hydrolyzed by DIPPs, but by competing with the active site, they act as inhibitors of PP-InsP hydrolysis [25,26,35]. Fiedler and colleagues showed that 1-PCP-InsP_7_ is an 80-fold more potent inhibitor of DIPP activity than is 5-PCP-InsP_7_ [26]. Such data are consistent with the aforementioned observation that DIPP prefers to hydrolyze 1-InsP_7_ over 5-InsP_7_. It is noteworthy that these biochemical assays were performed in media containing the Mg^2+^ that is necessary for activity. In contrast, Mg^2+^ was absent when Potter and colleagues [35] assessed apparent ligand affinities for DIPP by using differential scanning fluorimetry. Under such conditions, the apparent K_d_ values for 1-InsP_7_ and 5-InsP_7_ are very similar, while the apparent K_d_ value for 5-PCP-InsP_7_ is 3 to 4-fold lower than that for 1-PCP-InsP_7_ [35]. The fact that PCP versions of PP-InsPs recapitulate this Mg^2+^-dependence for DIPP’s preferential apparent affinity for 1-InsP_7_ over 5-InsP_7_ is testimony to their value as bioisosteres.

## 6. Application of Metabolically-Stable PP-InsP Analogues in Studies of Protein Pyrophosphorylation

By definition, metabolically-stable analogs of PP-InsPs cannot participate in phosphoryl transfer reactions; in fact, the PCP-analogues inhibit protein pyrophosphorylation, which presumably reflects their competitively inhibiting the natural PP-InsP from binding to the protein [26]. Such a phenomenon speaks to the pyrophosphorylation event requiring a specific binding event that could enforce PP-InsP specificity [26]. That is another valuable conclusion to be drawn from the use of these analogs.

Metabolically-stabilized derivatives of PP-InsPs (like PP-InsPs themselves) cannot penetrate into intact cells. Thus, to date, the use of these analogs to probe the possibility of protein pyrophosphorylation has been restricted to cell-free, in vitro assays. For example, there has been an investigation into the roles of PP-InsPs in regulating phosphorylation of interferon regulatory factor 3 (IRF3) [36]. This is a key step in inducing cellular interferon-β1 expression in response to viral infection. It was found that IRF3 phosphorylation in vitro is stimulated by 1-InsP_7_ and/or InsP_8_ [36]. Since the corresponding PA-analogues of 1-InsP_7_ and InsP_8_ proved to be inactive in this assay, it was concluded that the underlying mechanism for IRF3 activation likely involved protein pyrophosphorylation.

Another example has been a study of PP-InsP-mediated regulation of vesicle trafficking driven by cytoplasmic dynein 1 [37]. The latter is an important microtubule-based motor in many eukaryotic cells. In vitro, the dynein intermediate chain (IC), a non-catalytic dynein subunit, was shown to be pyrophosphorylated by 5-InsP_7_ on its N-terminus, which in turn promotes dynein-IC binding to the p150Glued subunit of dynactin [37]. Notably, 5-PCP-InsP_7_ was considerably less effective at enabling these protein-protein interactions [37], consistent with the idea that protein pyrophosphorylation is required.

## 7. Protein Stability

Potter and colleagues have used differential scanning calorimetry to show that the binding to DIPPs of either 1-PCP-InsP_7_ or 5-PCP-InsP_7_ elevates the protein’s thermal transitions temperature—which reflects increased protein stability—to almost the same degree as the corresponding PP-InsPs [35]. The authors posited that any protein stabilization by metabolically-stable PCP-InsPs might facilitate the formation of tractable crystal/ligand complexes for X-ray structural analysis.

## 8. Interrogation of the 5-InsP_7_ Interactomes

Fiedler’s group has prepared affinity resins to which 5-PCP-InsP_7_ has been immobilized by attachment of a linker to the 2-phosphate. This metabolically-resistant ligand has been deployed to search for 5-InsP_7_ binding partners in lysates of *S. cerevisiae* [38]. This particular application for the PCP-analogue yielded several interesting conclusions. Firstly, gene ontology analysis of the ‘hits’ showed a significant over-representation of proteins with bioenergetically important roles, such as nucleotide metabolism, glucose metabolism, and the energetically-intensive process of ribosome biogenesis. The nature of this cohort dovetails with the overarching concept that PP-InsPs play multiple roles at the interface of signaling and metabolic homeostasis [1].

This work was also informative because of the two separate screening conditions that were used: One contained 1 mM exogenous Mg^2+^, and in a second screening, endogenous Mg^2+^ was chelated by the addition of EDTA [38]. Similar numbers of proteins were identified in each screen (98 and 84, respectively), but only 21 overlapped both datasets. This is a singular observation that speaks to the existence of two structurally- and operationally-distinct PP-InsP binding sites. The authors speculated that Mg^2+^-independent binding could largely depend upon basic, positively charged amino-acid residues, whereas Mg^2+^-dependence may reflect the previously-demonstrated absolute dependence upon the cation for non-enzymic pyrophosphorylation of phosphor-Ser within an acidic context [39,40].

The 5-PCP-InsP_7_ affinity resin has also been used to screen for binding partners in cell extracts of the parasite, *Trypanosoma cruzi* [41]. As is the case for yeast extracts (see above), proteins involved in ribosome biogenesis and nucleotide turnover were both over-represented in the list of captured proteins. One of the novel targets, an uncharacterized protein, which is a putative phosphoribosyl pyrophosphate synthase, was found to contain a candidate SPX domain, which was recently shown to be a widespread PP-InsP binding module [42]. A novel pyrophosphorylation target was also identified and validated: a hypothetical choline/carnitine *o*-acetyltransferasedomain containing protein. Further work is needed to illuminate the biological significance of 5-InsP_7_ interactions with these proteins.

Fiedler and colleagues found that only 22 of the 5-PCP-InsP_7_ binding proteins in their yeast cell extracts (approximately 20% of the total) were *not* enriched in parallel screens performed with bead-immobilized InsP_6_ [1,38]. In discussing this observation, the authors asked how the other 80% of their ‘hits’ could perform as a specific regulatory molecule for 5-InsP_7_ in vivo, given that this PP-InsP has a total cellular concentration that is only 2–5% that of InsP_6_. This is a conundrum that permeates the field; other studies have also described binding proteins that show similar affinities towards PP-InsPs and InsP_6_ (e.g., [42]). Perhaps in part, the answer is provided by several separate lines of evidence—albeit indirect in nature (summarized in ref [2])—that indicate much of the cell’s complement of InsP_6_ is not free in the cytoplasm but instead may be accumulated into a number of cellular compartments, in which it cannot compete for PP-InsP binding sites.

In addition, Fiedler’s group speculated that there was another factor that contributed to their identification of only a small number of binding proteins with specificity for 5-PCP-InsP_7_ over InsP_6_: if a protein requires a 2-phosphate monoester for efficient binding, then its selectivity may be compromised by linker attachment at that position. This consideration prompted the group to subsequently synthesize triplexed affinity reagents – a mixture of linker attachments at either the 1-, 2- or 3-positions [43]. By using these more advanced reagents to screen lysates from HEK293 and HCT116 cells, the laboratory identified a 3-fold higher number of proteins exhibiting specificity for 5-PCP-InsP_7_ over InsP_6_, compared to their earlier study with yeast lysates. Among new potential PP-InsP binding proteins that should prove interesting to pursue are the kinase LKB1, which phosphorylates and regulates AMPK, and several enzymes that participate in purine metabolism; these are interesting targets in the context that PP-InsPs act as metabolic regulators.

## 9. Delivery of PP-InsPs and PP-InsP Analogues into Live Cells Using Nanocarriers

The ability to deliver an intracellular signal into cells can directly facilitate studies into its biological actions. However, the anionic nature of polyphosphates prohibits them from crossing the plasma membrane [44]. There are workarounds to this barrier, but several are unsatisfactory because they are physically or biochemically damaging, such as microinjection, electroporation, or chemical transfection (in any case, Lipofectamine 2000 does not efficiently transfer PP-InsPs into cells [45]). To circumvent these restrictions, the negative charges can be ‘hidden’ by incorporating the polyphosphates into biocompatible nanoparticles that can be accumulated into cells by endocytosis. For example, liposomes have been used to deliver a number of polar molecules into cells: microRNAs [46], siRNA [47], antisense DNA [48], and ATP [17,49]. We have recently adapted this technique in order to transfer PP-InsPs into cells; the cargo was spontaneously released during a 3–4 h loading protocol [50,51]. The mechanism of intracellular release appears to involve the fusion of the endocytosed liposomes with the endosomal and/or lysosomal membranes, causing the cargo to be ejected into the cytoplasm [52].

For example, these methods supported the discovery that 1,5-InsP_8_ regulates a cellular pathway for Pi efflux that is dependent upon Xenotropic and Polytropic Retrovirus Receptor 1 (XPR1). It was experiments with PPIP5K^-/-^ cells that initially led us to this idea that the Pi efflux pathway is licensed by 1,5-InsP_8_ [50]. To interrogate this genetically-based conclusion with an independent approach, we set out to complement the phenotype using our liposome delivery method. We delivered metabolically-stable 1,5-PCP-InsP_8_ into cells, and this rescued Pi efflux in a dose-dependent manner; delivery of either 1-PCP-InsP_7_ or 5-PCP-InsP_7_ was ineffective [50].

The relatively slow (3–4 h) liposomal delivery protocol described above has the disadvantage that it is not so useful for the delivery of the natural PP-InsPs; due to their rapid metabolism, it is hard to distinguish the functions of the original cargo from those of its metabolites. To ameliorate this restriction, we recently described the loading into populations of cultured cells of thermosensitive liposomes that only become permeable when the temperature exceeds 37 °C [53]. These liposomes are also doped with a photothermal, near infra-red carbocyanine dye, and can be induced to ‘melt’ and release their cargo within 5 min of illumination of the cultures by a high-power, near infra-red light-emitting diode. We used this methodology to deliver either 1,5-InsP_8_ or 1,5-PCP-InsP_8_ into HCT116 cells; both enhanced the rate of cellular Pi uptake to the same extent [53]. These quantitatively equivalent effects of 1,5-InsP_8_ and 1,5-PCP-InsP_8_ in a cell-based bioassay testify to the value of the PCP analog as a functional bioisostere when studying rapid signaling events that do not involve protein pyrophosphorylation.

In a more recent study, we pursued the discovery that DIPP1 not only hydrolyzes PP-InsPs such as 5-InsP_7_, but also cleaves the 5′-end 7-methylguanosine cap of a specific subset of mRNAs, under the guise of its NUDT3 pseudonym [54]. Decapping exposes the mRNA 5′-end to exonuclease-mediated degradation; this decay pathway plays critical roles in early animal development, cell growth and proliferation, immune response, and mRNA quality control [55]. We posited that 5-InsP_7_ might competitively inhibit mRNA decapping, and thereby stabilize the DIPP1-sensitive transcripts. This proposal was confirmed by liposome-mediated delivery of 5-PCP-InsP_7_ into HCT116 cells; this procedure elevated the levels of several DIPP1 mRNA substrates [51].

In this context, it is notable that 5-InsP_7_ levels fluctuate in response to the bioenergetic status of the cell (i.e., ATP levels) [56]; this homeostatic process underlies the role of 5-InsP_7_ in mediating nutrient-stimulation of insulin secretion from pancreatic β-cells [56]. These observations rationalize 5-InsP_7_ as being a rheostatic signaling molecule that protects bioenergetic health in the face of environmental challenges [1,6]. Thus, our delivery of PCP-InsP_7_ into cells using liposomes has unveiled an epitranscriptomic control process; we have shown how 5-InsP_7_ acts in a molecular pathway by which mRNA structure and stability can be modified in response to fluctuations in the extracellular environment.

Inhibition of 5′-decapping is associated with P-body accumulation [57,58]; the latter are highly dynamic, membrane-less biomolecular condensates that sequester certain mRNAs [59]. While the original concept that P-bodies regulate mRNA decay continues to be pursued [58,60], these organelles are now recognized as important sites for sequestration and storage of mRNAs away from the translating pool [61]. We have shown that liposome delivery of 5-PCP-InsP_7_ into cells elicits a >3-fold increase in the numbers of P-bodies per cell [51]. We are currently investigating if this effect on P-body dynamics is either a secondary consequence of the elevated levels of stabilized transcripts or a separate function for 5-InsP_7_ (e.g., perhaps through electrostatic interactions with mRNA, just as polyvalent interactions between arginine and RNA assist the phase separation process [62]). As for the ultimate fate of P-body enriched mRNAs, it has been proposed that these transcripts can be rapidly reintroduced into the translating pool, for example, in response to certain environmental cues [58,63,64].

**Figure 4:**
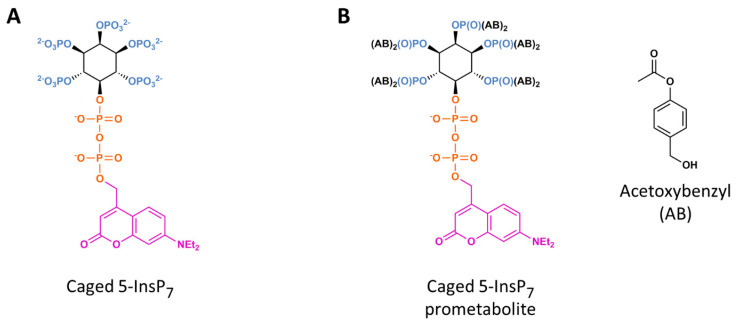
The structure of a (**A**) caged 5-InsP_7_ and (**B**) its ‘prometabolite’.

Alternative nanocarriers for PP-InsPs have been described, such as guanidinium-rich oligocarbonate transporters, which, like the liposomes described above, enter cells by the endocytic pathway and spontaneously release their cargo within approximately 4 h [45]. The latter study by Jessen and colleagues is also significant because it described the first synthesis and application of a ‘caged’ PP-InsP (Figure 4A and see Section 10 for further discussion).

## 10. Caged and Cell-Permeant PP-InsPs

Jessen and colleagues produced caged 5-InsP_7_ by adding [7-(diethylamino)coumarin-4-yl]methyl (DEACM) to the 5-β-phosphate (Figure 4A). The molecule was delivered into cells using guanidinium-rich oligocarbonate transporters [45]. This method offers a dramatic improvement to the temporal and spatial resolution of PP-InsP signaling. Uncaging was accomplished by cell exposure to a 30-s pulse from a 375 nm UV laser—the first example of controlled 5-InsP_7_ augmentation inside a living cell. Thus, it was possible to interrogate the hypothesis [65,66] that 5-InsP_7_ inhibits growth-factor mediated stimulation of AKT partly by competing with its PH domain (AKT^PH^) binding site for the signaling phospholipid, PtdIns(3,4,5)P_3_, which normally drives AKT translocation from the cytoplasm to plasma membrane, where it encounters upstream activating protein kinases. In their experiments, Jessen and colleagues uncaged 5-InsP_7_ after their AKT^PH^ construct had already been relocated to the plasma membrane by prior addition of growth factors [45]. A release of AKT^PH^ was observed after 5 min, and nearly completed by 15 min. The length of this time frame could indicate that it depends upon 5-InsP_7_ metabolism to 1,5-InsP_8_, but that seems unlikely because the latter is a significantly weaker AKT^PH^ ligand [67]. Perhaps instead there are physical constraints that slow down access of 5-InsP_7_ to AKT^PH^ that is already associated with PtdIns(3,4,5)P_3_. Indeed, even in vitro, 5-InsP_7_ is less potent at competing for the AKT^PH^ – bound PtdIns(3,4,5)P_3_ if the lipid is prebound to the protein [66].

Another approach to delivering PP-InsPs into cells is to use analogues that are inherently cell-permeant. This can be accomplished by masking the negative charges with lipophilic groups that are naturally removed once the compound has entered the cell; the application of this method to an inositol phosphate (InsP) was first described in 1994 [68]. Acetoxymethyl esters were deployed to mask the phosphate groups, while butyrate was added to the hydroxyls. The resultant, uncharged molecule can penetrate the plasma membrane, and once it is inside cells, intracellular esterases cleave the masking groups and yielded the native inositol phosphate. This technique has been adapted by Jessen and colleagues [69], such that caged 5-InsP_7_ was made cell-permeable by attaching acetoxybenzyl groups to the monophosphates and DEACM was added to the 5-β-phosphate (Figure 4B). Cell loading and removal of the acetoxybenzyl moieties was completed within 4 hr. Subsequently, UV-mediated uncaging of 5-InsP_7_ was found to block Ca^2+^ oscillations in the mouse pancreatic insulinoma (MIN6) cell-line, after a delay of 15 min [70]. In a subsequent study using caged 1,5-InsP_8_, its photorelease instantly stopped Ca^2+^ oscillations [71]. Thus, in the earlier study, it seems likely that the delay was due to 5-InsP_7_ phosphorylation to 1,5-InsP_8_. It can be anticipated that inhibition of Ca^2+^ signaling in β-cells by 1,5-InsP_8_ would inhibit exocytic insulin secretion [72], but this remains to be demonstrated.

On the other hand, Jessen and colleagues also described an apparently separate effect of 1,5-InsP_8,_ which, theoretically at least, could *promote* exocytosis, through PP-InsP’s interaction with synaptotagmin-like protein 4 (Slp-4)/granuphilin [71]. The latter is required for insulin granules to dock to the plasma membrane, but such vesicles are not fusion-competent until granuphilin is subsequently detached [73]. A mechanism for granuphilin release has previously been proposed: competition from cytosolic InsP_6_ for plasma-membrane associated PtdIns(4,5P)_2_ binding to granuphilin’s C2A and C2B binding domains [74]. However, 1,5-InsP_8_ was shown in Jessen’s study to also bind to each of these two domains in vitro (K_d_ = 0.6 and 5 µM, respectively [71], as compared to 1.8 and 21 µM for InsP_6_ [74]). Moreover, the caged 1,5-InsP_8_ proved useful in validating PP-InsP’s interaction with granuphilin in intact cells; a transfected and membrane-associated eGFP-tagged C2AB domain from granuphilin was found to redistribute into the cytoplasm following uncaging of the 1,5-InsP_8_ [71]. That is, 1,5-InsP_8_ was shown to be functionally active even in the presence of steady-state levels of InsP_6_. Nevertheless, further work is required to determine how the cell might coordinate the seemingly opposing effects upon insulin secretion that could arise from 1,5-InsP_8_ inhibiting Ca^2+^ signaling while also causing granuphilin relocalization.

## 11. FAM-5-InsP_7_

Recently, Jessen’s group synthesized a fluorescein-tagged analog of 5-InsP_7_ (FAM-5-InsP_7_) [51,53]. This, in itself, is notable for being the first description of the application of click chemistry to the synthesis of a PP-InsP analog, and the synthetic pathways that were involved [53] illuminate a gateway to the preparation of other novel molecules to facilitate further PP-InsP research. The value of this particular molecule is enhanced by the placement of the fluorescent tag onto the 5-β-phosphate, thereby imparting metabolic stability upon the normally labile diester phosphate [51].

We have used this new analog to validate our liposome delivery protocols [51,53] by exploiting the phenomenon of self-quenching that occurs when a fluorescent probe is present at high concentrations inside vesicles [75,76]. Thus, the gain of signal due to unquenching was used to follow the intracellular release of the FAM-5-InsP_7_ from the vesicles, predominantly into the cytoplasm, by using the tools of confocal microscopy and flow cytometry [51,53].

## 12. Conclusions

Based on the successes described above, we can confidently predict that further, dramatic developments in our understanding of PP-InsP biology will result as additional synthetic analogs provide information from inside intact cells and eventually in vivo. Two developments in particular appear close to fruition. First, photocage technology has improved to the point that the toxic effects of UV-mediated uncaging can be avoided by using photosensitive moieties that respond to visible light while retaining good water solubility [77]. Second, it has been proposed that one oxygen atom of the β-phosphoryl group of a PP-InsP can be replaced with a sulfur atom, so as to create an analog that can transfer a thiophosphoryl group to protein substrates for pyrophosphorylation [78]. Once transferred, the thiophosphate group can be derivatized and identified by appropriate antibodies. An InsP_7_-βS reagent could be delivered into cells using the nanoparticle delivery systems described above. These and other technical innovations will increase the opportunities available to those working in this unique field of signal transduction.

## Appendix A

A Note Concerning Inositol Pyrophosphate (PP-InsP) Nomenclature: The literature in this field has a divergent usage of abbreviations to denote individual inositol pyrophosphates and their analogues, accompanied by dueling opinions as to which system is the more accessible to a multidisciplinary audience; one approach to nomenclature is systematic in nature, whereas the other is more colloquial. The latter is the option taken in the text of the current study. Nevertheless, each of the corresponding systematic abbreviations are given here in parentheses, along with full definitions: 1-InsP_7_ (1-PP-InsP_5_) = 1-diphospho-*myo*-inositol 2,3,4,5,6-pentakisphosphate; 5-InsP_7_ (5-PP-InsP_5_) = 5-diphospho-*myo*-inositol 1,2,3,4,6-pentakisphosphate; 1,5-InsP_8_ (1,5-[PP]_2_-InsP_4_) = 1,5-bis-diphosphoinositol 2,3,4,6-tetrakisphosphate; 5-PP-InsP_4_ (also 5-PP-InsP_4_) = 5-diphospho-*myo*-inositol 1,3,4,6-pentakisphosphate; 1-PCP-InsP_7_ (1-PCP-InsP_5_) = *myo*-inositol 1-methylenediphosphonate-2,3,4,5,6-pentakisphosphate; 5-PCP-InsP_7_ (5-PCP-InsP_5_) = *myo*-inositol 5-methylenediphosphonate-1,2,3,4,6-pentakisphosphate; 1,5-PCP-InsP_8_ (1,5-PCP-InsP_4_) = *myo*-inositol 1,5-bis(methylenediphosphonate)-2,3,4,6-tetrakisphosphate; 1-PA-InsP_7_ (1-PA-InsP_5_) = *myo*-inositol 2,3,4,5,6-pentakisphosphate-1-phosphonoacetate; 5-PA-InsP_7_ (5-PA-InsP_5_) = *myo*-inositol 1,2,3,4,6-pentakisphosphate-5-phosphonoacetate; 1,5-PA-InsP_7_ (1,5-PA-InsP_5_) = *myo*-inositol 2,3,4,6-tetrakisphosphate-1,5-bis(phosphonoacetate).
